# Simultaneous percutaneous transcatheter aortic valve replacement and endovascular abdominal aortic aneurysm repair in a high risk patient with hostile aortic neck, a case report

**DOI:** 10.1186/s13019-015-0392-9

**Published:** 2015-12-12

**Authors:** Dimitrios Koudoumas, Vijay Iyer, Richard G. Curl

**Affiliations:** 1 SUNY at Buffalo, Department of Vascular Surgery, Buffalo General Medical Center, Gates Vascular Institute, 100 High Street, B-7, Buffalo, NY 14203 USA; 2 SUNY at Buffalo, Department of Interventional Cardiology, Buffalo General Medical Center, Gates Vascular Institute, Buffalo, NY USA

**Keywords:** Aortic stenosis, TAVR, Abdominal aortic aneurysm, EVAR

## Abstract

**Background:**

Abdominal aortic aneurysm (AAA) can be a potential life threatening condition if left untreated. Total endovascular techniques to approach aortic aneurysms have extended management options and enabled patients who are unfit for open surgery to undergo repair. Transcatheter aortic valve replacement is increasingly used to treat patients with severe symptomatic aortic stenosis, who once were considered high risk for traditional open aortic valve replacement.

**Results:**

Herein we report the complete simultaneous treatment of an infrarenal AAA with hostile neck and severe aortic stenosis in a patient deemed high risk for surgical repair.

**Conclusion:**

Advances in catheter based endovascular technology have enabled physicians to approach patients with AAA and valvular pathology even with multiple comorbidities that otherwise would be poor surgical candidates, even in the presence of challenging anatomic considerations and various comorbidities.

## Background

The incidence of severe aortic valvular disease in the abdominal aortic aneurysm (AAA) population is largely unknown. Previously open simultaneous aneurysm repair and cardiac surgery has been advocated in highly selected patients with acceptable outcomes [1]. Endovascular aortic aneurysm repair (EVAR) has became the mainstay of treatment for the majority of AAA, in patients with favorable neck anatomy, and has enabled patients who are unfit for surgery to undergo repair with acceptable results [2]. Transcatheter aortic valve replacement (TAVR) has been increasingly used in patients with severe symptomatic aortic valve stenosis (AS), who are considered at high risk for traditional open surgical aortic valve replacement (AVR). In this case report, we describe the complete and combined percutaneous endovascular repair of an infrarenal AAA and AS in a patient with hostile aortic neck. Formal informed consent was obtained from the patient prior to submitting the case for publication.

## Case presentation

A 74-year-old male with diabetes, chronic kidney disease, chronic obstructive pulmonary disease not on home oxygen therapy, cerebrovascular disease, status post left superficial temporal to middle cerebral artery bypass, left carotid endarterectomy and subsequent stent placement, 3-vessel coronary artery disease status post coronary artery bypass graft and non small cell right lung cancer stage IIIA status post induction chemo-radiation with response and downstage to stage IIA, was referred for increasing shortness of breath on minimal exertion for the last 6 months and abdominal pain. Of note he had an expanding infrarenal abdominal aortic aneurysm measuring 5 cm on recent computed tomography angiography (CTA), which had increased from 4 cm in 6 months. Echocardiogram showed severe AS with mean gradient of 43 mmHg, aortic orifice size of 0.48 cm^2^, mild aortic and mitral insufficiency, ejection fraction of 30–35 % with hypokinesis of anterior apical region and mild pulmonary hypertension. Left heart catheterization revealed complete patency of all five by-pass grafts. In the presence of his complicated medical and surgical history he was deemed high risk candidate for surgical aortic valve replacement with an STS score calculated at 6.2 % and decision was made to proceed with simultaneous TAVR and EVAR to address both the AS and AAA.

**Fig. 1:**
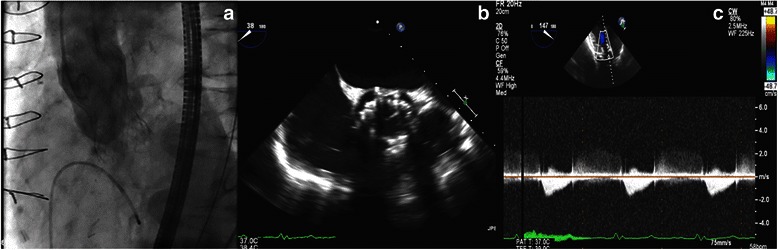
**a** Valve implantation. **b** TEE post implantation and dilatation. **c** Transgastric Aortic Valve gradient

Under general anesthesia, double Proglide/Preclose technique was used to access both femoral arteries. A 31 mm CoreValve (Medtronic Inc., Minneapolis, Minnesota) was deployed across the aortic annulus (Fig. 1a) delivered through a 18-F sheath from the left groin. Under transesophageal control mild-to-moderate paravalvular leak was noted and the valve was post dilated with a 24 mm aortic valvuloplasty balloon. This lessened the degree of aortic insufficiency and by aortic root angiography was noted to be trivial with excellent positioning of the valve on the annulus (Fig. 1b). Transgastric view confirmed minimal gradient across the valve (Fig. 1c).

**Fig. 2:**
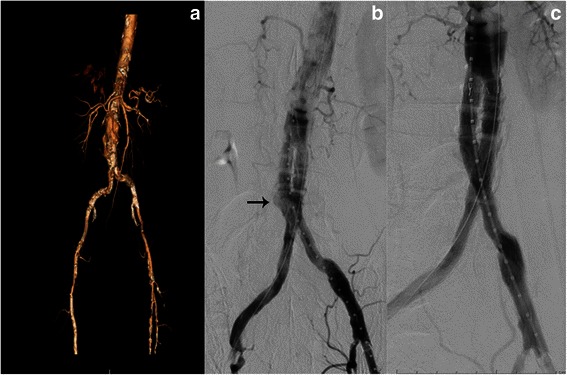
**a** 3D reconstruction CT preoperatively. **b** Type 3 endoleak (*arrow*). **c** Completion angiography

Direct review of the angiographic imaging along with preoperative review of the CTA and CT reconstruction (Fig. 2a) that had depicted a 14 mm proximal neck, deemed the patient good candidate for a 20 mm Ovation Prime Abdominal Stent Graft System (TriVascular, Inc., Santa Rosa, CA), which was placed uneventfully. Completion angiography revealed a probable type 3 endoleak (Fig. 2b, arrow) originating in the overlap of the limbs and the main body that was approached with serial balloon dilations. Repeat completion angiogram showed resolution of the type 3 endoleak (Fig. 2c) and complete exclusion of the AAA. The patient tolerated the procedure well and was extubated in the operating. He had an uneventful hospital stay and was discharged home on postoperative day 3.

At his 1-week follow up, the patient felt significantly better with NYHA I symptoms. Echocardiographic follow up revealed no significant aortic regurgitation and no transvalvular gradient, which persisted in 3-month follow up study. The arteriotomy sites were well healed with excellent peripheral perfusion.

## Discussion

Traditionally, aortic valve replacement followed by AAA repair is planned in patients with severe aortic stenosis and concomitant AAA, however, simultaneous repair has been advocated in patients with extremely large or symptomatic AAA, mainly due to concerns regarding increased rupture risk after open heart surgery, likely due to mobilization of matrix metalloproteinases [3, 4]. In the last decade, AVR followed by EVAR has been used to decrease the intensity of the simultaneous repair in such patients, and most recently, simultaneous endovascular repair has been reported [5, 6]. This approach enabled our extremely high-risk patient with symptomatic AAA and hostile neck to be treated successfully.

Indications for combined approach could be – but not limited to - poor surgical candidates for open cardiac surgery based on STS or EuroSCORE, previous open cardiac surgery, multiple comorbidities, advanced aged, favorable neck anatomy and good access vessels.

Two variations of simultaneous approach may exist. Performing the EVAR followed by the TAVR may eliminate potential dissections caused by the valve device and prevent potential rupture of the aneurysmal sac or dissection due to increased blood pressure following prosthetic valve implantation and relieve of the AS. Presence of increased clot burden within the aneurysmal sac and potential risk of distal embolization with wire manipulation and TAVR device may serve as indication to perform the procedure in this orientation. On the other hand the presence of CHF with low EF and thus smaller variations and increase in blood pressure may serve as an indication to perform first the TAVR and subsequently the EVAR, since reverse cardiac remodeling and recovery of ventricular function does not happen immediately postoperatively [7]. Also, the absence of the EVAR body and/or limbs may hamper delivery of the TAVR device, which can be avoided in this sequence.

An unfavorable neck anatomy is the most frequent cause of exclusion from EVAR [8]. Length < 15 mm, angulation between 50° and 60°, diameter ≥26 mm, the presence of calcification, circumferential thrombus, bulge or reverse taper neck are considered anatomic hostile neck characteristics [9]. The Ovation Prime Abdominal Stent Graft System (TriVascular, Inc., Santa Rosa, CA) has been recently reported to treat patients with narrow access vessels and short proximal necks, without sacrificing patient safety or device effectiveness [10]. Small number of case reports has been reported in the literature of simultaneous EVAR and TAVR, all with favorable neck anatomy [11–13].

It has been proposed that survival is mainly determined by the stage of lung cancer in patients with concomitant AAA and lung cancer, and treatment should be highly individualized [14]. Our patient had rapidly enlarging symptomatic AAA and his lung cancer was deemed responsive to chemotherapy. Published data suggest that patients with AAA that undergoing chemotherapy may be at increased risk for aortic rupture [15]. We thought that the most efficient and safest way to address the AAA was simultaneous repair, once he was deemed appropriate for TAVR. Recent published data suggested that active treatment for lung cancer is a prognostic factor for late post EVAR mortality and decision to pursue intervention should be individualized [2].

Herein we present an unusual case of simultaneous and complete catheter based therapy to approach a patient with simultaneous AS and AAA with hostile neck anatomy and favorable prognosis from a lung cancer perspective.

## Conclusion

Advances in catheter based endovascular technology have enabled physicians to approach patients with AAA and valvular pathology even with multiple comorbidities that otherwise would be poor surgical candidates, even in the presence of challenging anatomic considerations and various comorbidities.

## Consent

Written informed consent was obtained from the patient for publication of this case report and any accompanying images. A copy of the written consent is available for review by the Editor-in-Chief of this journal.
